# Methamphetamine Intoxication and Perioperative Complications Following Orthopaedic Surgical Procedures

**DOI:** 10.7759/cureus.19082

**Published:** 2021-10-27

**Authors:** Erin P Murray, Leilani Mansy, Joshua T Lackey, Robert C Link, Sean Bonanni, Amelia A Sorensen

**Affiliations:** 1 Orthopaedic Surgery, University of Missouri - Kansas City, Kansas City, USA

**Keywords:** outcome studies, delay in surgery, ortho, ards (acute respiratory distress syndrome), perioperative complication, orthopaedic surgery, methamphetamine use

## Abstract

Background

Methamphetamine use is increasing in prevalence. There is a theoretical increased risk of complication postoperative due to catecholamine depletion. When presented with an urgent surgical problem, there are little data to help counsel the patient on the risks of undergoing surgery in the setting of a positive methamphetamine test result.

Aims and objectives

The aim of this study was to examine the perioperative complication rate for patients who underwent emergent orthopaedic procedures in the setting of a positive methamphetamine drug screen. Additional data were collected in an attempt to further stratify risk factors for perioperative complications in this patient population.

Design and methods

A retrospective case series of 110 patients. Patients were identified by querying the medical record for patients with a positive methamphetamine result within 24 hours of the surgery start time. Data were collected on each patient, including the nature of the surgery, the type of injury sustained, disposition from the operating room, among other data points. The primary outcome was the presence of a perioperative cardiopulmonary complication, as determined by a new diagnosis made in the chart. The secondary outcome was whether the patient needed an increased level of care postoperatively.

Results

Of the 110 charts reviewed, three patients sustained complications during their hospitalization; an overall complication rate of 2.7%. One patient developed acute respiratory distress syndrome (ARDS), while two others developed surgical site infections. Of the 19 patients who went to the intensive care unit (ICU) postoperatively, none were because the patient required a higher level of care than the preoperative level.

Conclusions

Patients who underwent emergent surgical intervention in the setting of a positive methamphetamine drug test had a low complication rate. While the dogma is to delay surgery in the setting of methamphetamine use, the true risk of undergoing surgery in this setting is not fully understood. We advocate for continued research in this poorly studied group of patients. Larger studies will need to be done in order to fully understand the risks associated with operating in the setting of a positive methamphetamine drug screen.

## Introduction

Methamphetamine use has become increasingly common in society [1]. When these patients present with urgent surgical problems, there is minimal data to guide surgeons' and anesthesiologists' efforts to counsel patients regarding the risks of surgery. The majority of orthopaedic procedures fall into the intermediate-risk category, with an accepted perioperative risk of 1-5% [2]. There are little data, however, regarding the risk of intraoperative and perioperative complications in acutely intoxicated patients undergoing surgical procedures.

To the best of our knowledge, there has only been a single study that demonstrated a similar rate of perioperative complications between patients acutely intoxicated with methamphetamine and the accepted 1-5% perioperative risk after undergoing an orthopaedic surgical procedure [2,3]. The primary objective of this study was to determine the perioperative complication rate in patients undergoing orthopaedic surgical procedures within 24 hours of a methamphetamine-positive drug screen. The secondary objective was to evaluate if these patients required a higher level of care postoperatively.

## Materials and methods

After approval by the Institution Review Board of Truman Medical Center (IRB number 19-149), patients were identified by query of the electronic medical record from an urban level one trauma center. Inclusion criteria included: age >18 years old, drug screen positive for methamphetamine, and orthopaedic surgery start time within 24 hours of the positive drug screen result. Patients were excluded if they were less than 18 years of age at the time of surgery or if there was an incomplete electronic medical record. The database was queried for patients who met inclusion criteria and underwent surgery between the years 2010 and 2020. Ultimately 110 patients met inclusion criteria and their electronic medical records were reviewed. We recorded additional patient data including demographic information, smoking status, BMI, American Society of Anaesthesiologists (ASA) physical classification status, and airway type (endotracheal tube, laryngeal mask airway, LMA, local). Detailed information about the procedure including the location of injury (upper extremity, pelvis/acetabulum, hip/femur, knee/tibia, foot/ankle), type of procedure (irrigation and debridement, damage control, definitive fixation, fasciotomy, or spine decompression), and time of day the surgery occurred were also recorded.

The primary outcome measure was the perioperative complication rate. The medical record was reviewed for the remainder of the patients' hospitalization for perioperative complications including cardiovascular events such as stroke, myocardial infarction (MI), acute respiratory distress syndrome (ARDS), deep vein thrombosis (DVT), and pulmonary embolism (PE). Patient disposition from the operating room was recorded, including an increase in the level of care (i.e., floor to intensive care unit, ICU, status). All patients who were transferred to the ICU postoperatively were evaluated if they were extubated prior to leaving the operating room.

The number of patients included in our study was limited to those we were able to identify who underwent surgical intervention with a positive methamphetamine drug screen. Patients were identified using a search of the electronic medical record to identify patients with a positive methamphetamine drug screen, and subsequently excluded those who did not have a surgery start time within 24 hours with one of the staff orthopaedic surgeons at our hospital. We were not able to control for the timing of the last use of methamphetamine to surgery. We also did not distinguish between chronic methamphetamine use and an isolated episode of use. Additionally, patients may have been excluded from our search who had surgical intervention but the drug screen was done after surgery, or if the primary surgeon listed in the medical record was not an orthopaedic surgeon, such as in cases of trauma patients taken emergently to the operating room.

## Results

A total of 110 patients met the inclusion criteria. There were 77 males and 33 females. The average age was 40.58 years (range 18-70 years). Of the 110 patients in the study, 76 identified as current tobacco cigarette smokers. The majority of patients were classified as ASA 3 (80 patients) or ASA 2 (22 patients); 42 patients had BMI under 25, while 65 patients had a BMI over 25. The BMI was unknown in three patients. Eight-two patients were Caucasian, 17 were African American, two were Hispanic, and nine were either unknown or chose not to disclose their race (Table 1).

**Table 1:** Demographics ASA: American Society of Anaesthesiologists physical classification status, BMI: body mass index

Gender	
Male	77
Female	33
Age (years)	40.58
Range	18–70
Smoking status	
Smoker	76
Non-smoker	20
Previous smoker	4
Unknown	10
Race	
White/Caucasian	82
African American	17
Hispanic	2
Chose not to disclose/other	7
Not documented	2
ASA	
1	0
2	22
3	80
4	8
BMI	
<18.5	2
18.5–25	40
25–30	41
30+	24
Unknown	3

The drug screen was evaluated for every patient. In 64 patients, methamphetamine was the only positive result. Some drug screens were positive for more than one substance, but we did not exclude these patients to make our results more generalizable. Other concomitant positive results included marijuana (53 patients), cocaine (13 patients), and phencyclidine (6 patients). The anatomic location of the surgery was variable. In 47 patients, this involved the upper extremity.

This compared to 26 patients with knee/tibia, 23 patients with hip/femur, 11 patients with foot/ankle, two patients with pelvis/acetabulum, and one patient with spine pathology. The pathology itself was also recorded. Seventy-two patients who required emergent surgical intervention were related to fractures, with 35 of those being open fractures. Thirty-five patients had musculoskeletal infections requiring debridement in the operating room. Three patients had a progressive neurologic deficit requiring surgical decompression (Table 2).

**Table 2:** Drug screen results and pathology MSK infection: musculoskeletal infection

Drug screen	
Only positive for Meth	64
Other positives identified	
Marijuana	53
Cocaine	13
PCP	6
Pathology	
Fracture	72
Open fracture	35
Closed fracture	37
MSK infection	35
New neurologic deficits	3
Other	
Body location	
Upper extremity	47
Pelvis/acetabulum	2
Hip/femur	23
Knee/tibia	26
Foot/ankle	11
Spine	1

The majority of surgical interventions (67 of 110) occurred during 0600-1359, followed by 1400-2159 (38 of 110). Only 10 of the surgeries were performed under local anesthesia with sedation, with the remaining having either an LMA or endotracheal tube placed. Of the 110 surgical procedures, 90 patients were transferred to the floor after recovering from anesthesia. There were 19 patients who went to the ICU postoperatively and one patient was transferred to the progressive care unit (PCU). Of the patients transferred to the ICU postoperatively, seven remained intubated after surgery was complete. All patients in either the ICU or PCU following surgery were at the same level of care prior to surgery. No patients required an increased level of care postoperatively (Table 3).

**Table 3:** Procedure details I&D: irrigation and debridement, LMA: laryngeal mask airway, MAC: monitor anesthesia care, PCU: progressive care unit

Procedure type	
I&D (not for open fracture)	49
Damage control	7
Definitive fixation	60
Nail	18
Plate	22
Other (pin, suture, etc)	20
Spine decompression	1
Fasciotomy	1
*Eight patients underwent multiple procedures, total procedures in this study 118	
Surgery start time	
06:00–13:59	67
14:00–21:59	38
22:00–05:59	5
Airway type	
Endotracheal tube	63
LMA	33
Local MAC/local only	10
Not documented	4
Disposition from OR	
Float/PACU	90
PCU	1
ICU	19
Intraoperative death	0
Of the 19 patients to ICU	
Remained intubated after surgery	7
Extubated after surgery	12

Overall, the rate of perioperative complications was low. One patient developed ARDS on postoperative day eight. This patient has multiple risk factors for ARDS including pulmonary contusion and multiple long bone fractures, so it is difficult to determine what role methamphetamine use may have played. Two additional patients developed surgical site infections, both of which occurred greater than two weeks after the initial surgery. The overall complication rate was 2.7%, with a cardiopulmonary complication rate of 0.9%.

## Discussion

Acutely intoxicated patients commonly are injured via high-energy mechanisms, such as motorcycle and motor vehicle crashes, and are more likely to require urgent or emergent surgical intervention [1]. Previous studies have shown that methamphetamine-positive patients have approximately the same risk for an ICU admission when compared to the methamphetamine-negative patient, while others put this group at a slightly higher risk [1,4]. Pre-injury drug use has also been associated with longer hospital stays by Shymon et al., but they did not study if there was a delay in surgical intervention for that patient population [5]. 

The role of surgical intervention in acutely intoxicated patients is not known. While there is a theoretical increase in perioperative risk due to catecholamine depletion in these patients, there is little modern evidence to substantiate this claim beyond anecdotal or theoretical basis [6]. As Fischer et al. described, many of these guidelines have been based on very limited clinical data from studies conducted nearly 40 years ago [7].

More recent literature suggests that the lability seen in this patient population may be more directly related to the amount of resuscitation, and not the presence of a positive drug screen alone [8]. Clinical use of amphetamines in the treatment of attention deficit hyperactive disorder has also been studied and found to have no significant increased risk of cardiovascular events such as myocardial infarction, stroke, or sudden cardiac death [9]. This study provides objective data to further understand the risk of patients undergoing orthopedic procedures while acutely intoxicated on methamphetamine. 

To date, we were only able to identify one study regarding orthopedic procedures in acutely methamphetamine-intoxicated patients. Githens et al. conducted a retrospective review of 94 patients who had a positive drug screen within two days of the procedure [3]. In our study, we applied a stricter timeline of 24 hours from the time of the positive drug screen. The primary endpoint of their study was the presence of perioperative complications. Of the 94 patients studied by Githens et al., only two had perioperative cardiovascular complications, one of which was intraoperative death due to hypovolemic shock secondary to the traumatic injuries sustained [3].

In our study, one patient developed ARDS on postoperative day eight, however, it is difficult to attribute this to the methamphetamine alone. This patient had multiple other risk factors for ARDS including pulmonary contusions and multiple long bone fractures. Two additional patients developed surgical site infections after prolonged periods of hospitalization. No patients developed perioperative cardiovascular-related complications. While we examined the medical record for diagnosis such as myocardial infarction, stroke, hypertensive urgency, etc., we did not collect information on patients who were initiated on blood pressure medication in the hospital. Being a tertiary care facility, we have many patients who are transferred or present to our facility without available past medical records. Due to the retrospective nature of the study, it was difficult to determine whether blood pressure medications found in the medical record were resumed home medications, in-hospital treatment of baseline uncontrolled blood pressure, or started as a consequence of methamphetamine use prior to surgery among various reasons. The interpretation of the reasons would create a source of bias. In order to remain as objective as possible, we only included official diagnoses made in the patient's charts.

The perioperative complication rate from our study and that of Githens et al. both fall well within the expected 1-5% cardiac risk associated with orthopedic procedures according to the American College of Cardiology guidelines [2,3]. These small studies should still be taken into consideration carefully when considering the application to a patient in practice, as it would likely take a larger sample of patients before a definitive conclusion regarding the safety of surgery in methamphetamine-positive patients. Ultimately, a discussion between the surgical and anesthesia teams is needed in order to balance the consequences of delaying surgical intervention and the risk of anesthesia in the acutely methamphetamine-positive patient.

There were limitations of our current study. We did not account for the possibility of false-positive results, which can occur with numerous different medication interactions as described by Saitman et al. and Brahm et al., nor did we have confirmatory testing results for all patients [10,11]. We also did not differentiate between acute versus chronic methamphetamine use. Patients who are on chronic methamphetamine use almost certainly have a different physiologic response compared to an isolated use, but the information available in the medical record often made the differentiation impossible. Finally, we only followed the patients during the immediate postoperative period until they were discharged from the hospital. It is possible that cardiovascular events occurred following discharge from the hospital.

## Conclusions

This study demonstrated similar perioperative complications between the methamphetamine-intoxicated patients and the accepted risk associated with orthopaedic procedures defined by the American College of Cardiology guidelines. While the sample size was relatively small, it emphasizes the need for additional research into this understudied field to determine what the true perioperative risk of this patient population is. Prospective studies may provide further insight into the true perioperative risk for this understudied patient population. The data from this study would support the conclusion that urgent surgical problems do not need to be delayed based solely on a positive methamphetamine drug screen.
